# Supplementary material for: Reconciling Mining with the Conservation of Cave Biodiversity: A Quantitative Baseline to Help Establish Conservation Priorities
Source: PLoS One. 2016 Dec 20;11(12):e0168348. doi: 10.1371/journal.pone.0168348 (PMC5173368; doi:10.1371/journal.pone.0168348)
Supplement: S1 Dataset — (ZIP) [file pone.0168348.s002.zip › Taxa/Serra Norte/SN_2013/N4E/Lista N4E-79.pdf]

CAVIDADE N4E-0079

Classe	Ordem	Fam/Outros	Gên/Outros	Espécie	S-U	U-S	Zon.
Arachnida	Acari	(Parasitengonina)		sp.2		X	P
Arachnida	Acari	Neothyridae	*Diplothyrus*	jovem		X	P
Arachnida	Amblypygi	Charinidae	*Charinus*	sp.1		2	P
Arachnida	Araneae	Araneidae	*Mangora*	sp.1	X	1	E
Arachnida	Araneae	Corinnidae		jovem	2	2	P
Arachnida	Araneae	Drymusidae		jovem		X	P
Arachnida	Araneae	Filistatidae		sp.1	X	X	E/P/A
Arachnida	Araneae	Ochyroceratidae	*Ochyrocera*	sp.3		X	P
Arachnida	Araneae	Pholcidae	*Leptopholcus*	sp.1		X	E
Arachnida	Araneae	Pholcidae	*Mesabolivar*	*aurantiacus*	X		
Arachnida	Araneae	Pholcidae	*Mesabolivar*	sp.1	X	X	E/P
Arachnida	Araneae	Scytodidae	*Scytodes*	*eleonorae*	3	5	E/P
Arachnida	Araneae	Segestriidae		jovem		X	P
Arachnida	Araneae	Theridiidae	*Theridion*	sp.1		X	E
Arachnida	Araneae	Theridiidae	*Theridion*	sp.4		X	P
Arachnida	Araneae	Theridiosomatidae	*Plato*	sp.1	X	X	E/P
Arachnida	Opiliones	Cosmetidae	*Roquettea*	*singularis*		1	E
Arachnida	Opiliones	Stygnidae	*Protimesius*	*gracilis*	1		P
Arachnida	Pseudoscorpiones	Cheiridiidae		jovem		X	P
Arachnida	Pseudoscorpiones	Chernetidae	*Spelaeochernes*	sp.1	X	X	E/P
Arachnida	Pseudoscorpiones	Chthoniidae	*Pseudochthonius*	sp.1		X	P
Arachnida	Schizomida	Hubbardiidae	aff. *Rowlandius*	sp.1		X	P
Diplopoda	Polydesmida	Paradoxosomatidae		sp.1		1	P
Diplopoda	Siphonophorida	Siphonophoridae		sp.1		X	P
Entognatha	Collembola	Cyphoderidae		sp.1	X		A
Entognatha	Collembola	Paronellidae		sp.4		X	P
Entognatha	Diplura	Campodeidae		sp.1	X	X	E/A
Insecta	Blattodea	Blattellidae		sp.5	1	5	P
Insecta	Blattodea	Blattidae		sp.5	13		P/A
Insecta	Blattodea	Polyphagidae		sp.1	15	14	E/P/A
Insecta	Coleoptera			jovem	X	X	E/P/A
Insecta	Diptera	Culicidae	*Culex*	sp.		X	E
Insecta	Diptera	Phoridae		sp.		X	E
Insecta	Diptera	Psychodidae	Phlebotominae	sp.	X		P
Insecta	Diptera	Tipulidae	Tipulinae	sp.	X	X	E/P
Insecta	Heteroptera	Reduviidae	Emesinae	sp.		1	P
Insecta	Heteroptera	Reduviidae	Reduviinae	sp.	6	6	E/P
Insecta	Homoptera	Cixiidae		jovem		X	E
Insecta	Hymenoptera	Formicidae	*Camponotus*	sp.2	X	X	E/A
Insecta	Hymenoptera	Formicidae	*Hypoponera*	sp.2		X	P
Insecta	Hymenoptera	Formicidae	*Pachycondyla*	sp.2	X	X	E/A
Insecta	Isoptera	Termitidae	*Nasutitermes*	sp.	X	X	E/P/A
Insecta	Lepidoptera	Noctuoidea		sp.1		2	E
Insecta	Lepidoptera	Noctuoidea		sp.5		1	E
Insecta	Lepidoptera	Tineoidea		sp.1		X	P
Insecta	Lepidoptera	Tineoidea		sp.2	X	X	E/P
Insecta	Lepidoptera	Tineoidea		sp.3	X	X	E/P
Insecta	Neuroptera	Mantispidae	*Plega*	sp.		1	E
Insecta	Orthoptera	Phalangopsidae	*Paraclodes*	sp.		10	E

Insecta	Orthoptera	Phalangopsidae	*Phalangopsis*	sp.	1	54	P/A
Insecta	Psocoptera	Psyllipsocidae		jovem	X	X	P
Insecta	Thysanura	Ateluridae		jovem		X	P
Insecta	Thysanura	Nicoletiidae		sp.1	X	X	E/A
Gastropoda	Pulmonata	Systrophiidae	*Happia*	sp.		X	E/P
Symphyla		Scutigerellidae		sp.		X	P
Amphibia	Anura	Leptodactylidae	*Leptodactylus*	cf. *vastus*	1		P
Amphibia	Anura	Strabomantidae	*Pristimantis*	cf. *fenestratus*	2		P
Mammalia	Chiroptera	Furipteridae	*Furipterus*	*horrens*	20		E/P
Mammalia	Chiroptera	Phyllostomidae	*Carollia*	*perspicillata*		1	E
Mammalia	Rodentia	Cricetidae	*Rhipidomys*	sp.	1	1	E/A
